# Supplementary material for: Metal-hydrogen systems with an exceptionally large and tunable thermodynamic destabilization
Source: Nat Commun. 2017 Nov 29;8:1846. doi: 10.1038/s41467-017-02043-9 (PMC5705672; doi:10.1038/s41467-017-02043-9)
Supplement: Supplementary file 1 — Supplementary Information [file 41467_2017_2043_MOESM1_ESM.pdf]

Supplementary Figures

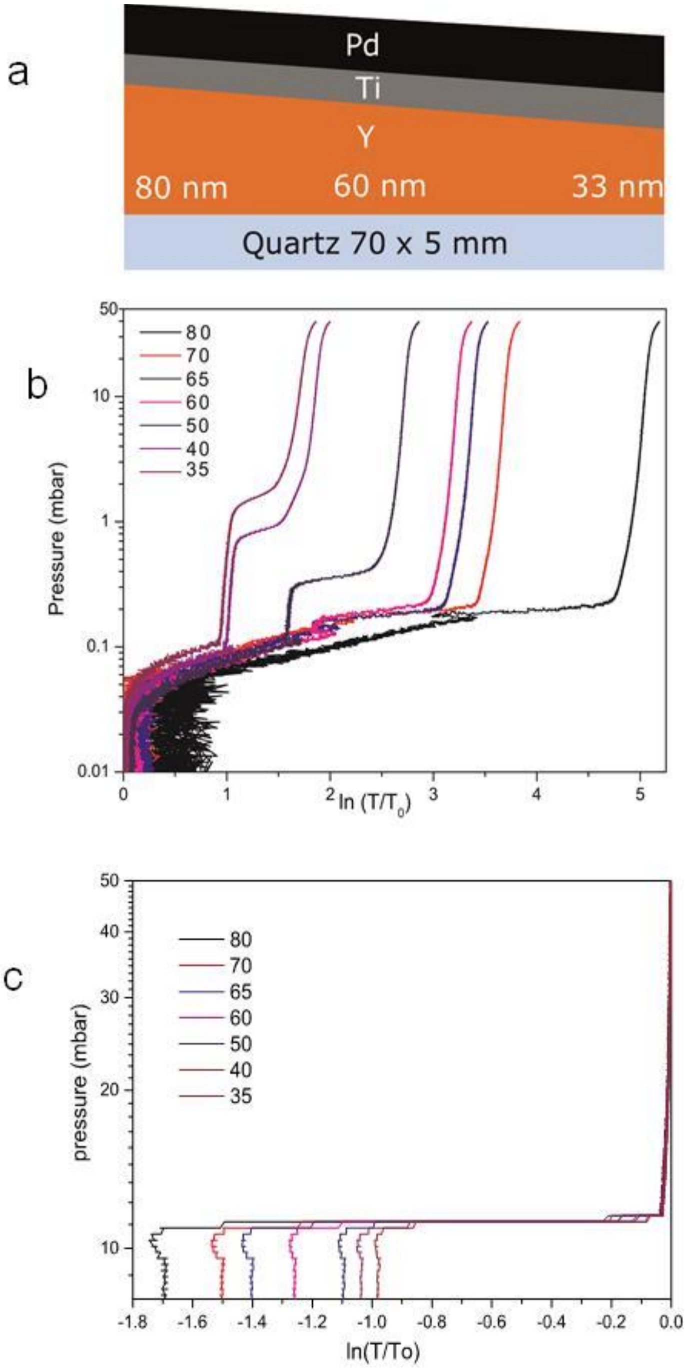

Supplementary Figure 1: (a) The configuration of a Y-Ti-Pd multilayer thin film deposited on a 70 mm x 5 mm quartz substrate. The Y thickness varies linearly from 33 nm to 80 nm along the 70 mm quartz substrate as shown, while Ti and Pd have a constant thickness of 5 and 15 nm respectively. (b) Pressure transmission isotherms (PTIs) for the first cycle hydrogen absorption measurement at room temperature (25 °C). The figure shows the PTIs of selected positions along the sample length and the thickness of the Y layer that corresponds to the PTI. It can be seen that the magnitude of the total change in transmittance (x-axis) decreases as the Y thickness (hence the total amount of H absorbed) decreases, in line with the Lambert- Beer- law (equation 4 in the main text). The increase in the plateau hydrogen pressure for $YH_2 \rightarrow YH_3$ transition with decreasing Y thickness is related to the difference in the interface energy per unit volume at the Y-Ti interface, as we previously reported for Mg based samples.[1] Clearly, the effects of the difference in thickness are small in comparison to the effects of Zr addition. (c) PTIs for the dehydrogenation of fully hydrogenated samples i.e. $YH_3 \rightarrow YH_2$ at 220 °C. The numbers on the legend indicates the Y thickness. Unlike for the hydrogenation experiment, the equilibrium dehydrogenation pressures do not depend on the thickness of the Y. Therefore the tunable (de)hydrogenation pressures observed in the YZr samples (Figure 1 b-d in the main manuscript) is unequivocally due to the presence of Zr. This is also supported by Supplementary Figure 4.

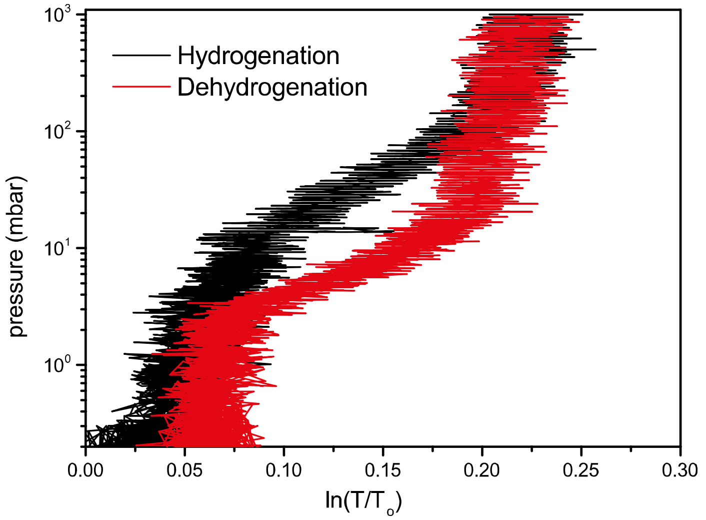

Supplementary Figure 2: Hydrogenography result showing the PTI for hydrogen absorption and desorption in the Zr-Ti-Pd multilayer thin film (60 nm Zr-5 nm Ti-15 nm Pd) at room temperature (25 °C). Comparing this PTI to that in Supplementary Figure 1 shows that the optical change due to hydrogen absorption in Zr is small compared to the optical change arising from hydrogenation of Y. The total change in transmittance observed here includes the contribution due to (de)hydrogenation of the Pd cap layer around 40 mbar.

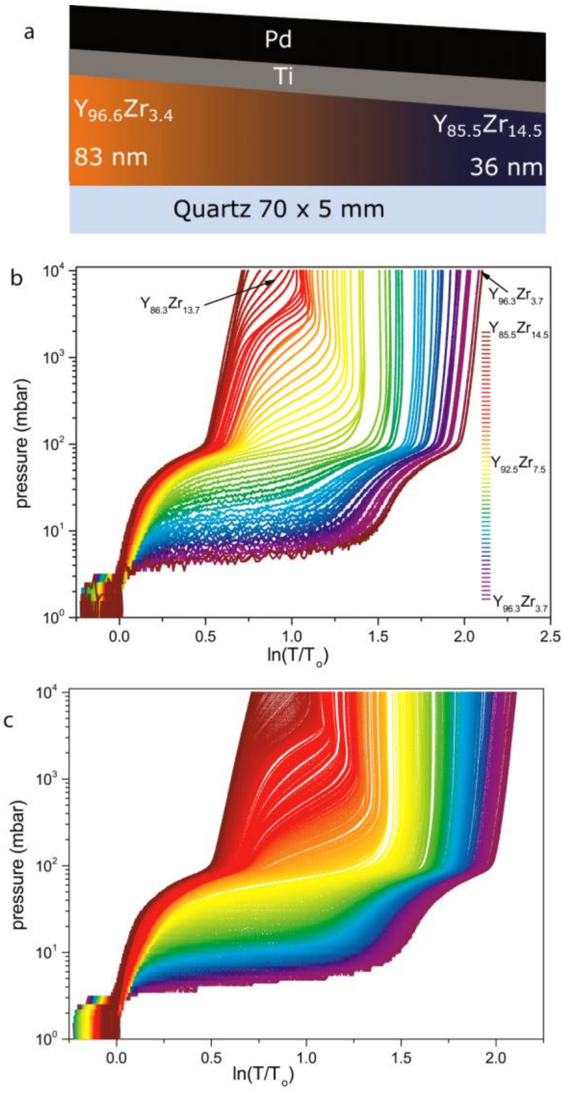

Supplementary Figure 3: (a) Configuration of an Y-Zr gradient thin film with 3.4-14.5 at.% Zr ($Y_{96.6}Zr_{3.4}$ - $Y_{85.5}Zr_{14.5}$). (b) 10th hydrogenation cycle at room temperature showing the PTIs of 50 positions along the sample, with each position/step or PTI corresponding to about 0.217 atom% increase in Zr , starting from $Y_{96.3}Zr_{3.7}$. It can be seen that the region of the sample with more than 13% Zr are not fully hydrogenated even at 10^4 mbar. (c) PTIs of 512 positions along the sample length, hence each step or PTI corresponds to about 0.0217 at.% increase in Zr concentration. Clearly, the plateau pressure can be continuously and precisely tuned from 3 mbar to 10,000 mbar, which is the safety limit of our hydrogenography set-up. The increased tilting of the isotherm at higher Zr concentration is due to the fact that the hydrogen pressure is increased logarithmically from 1 to 10,000 mbar, resulting in a higher step size at higher pressures.

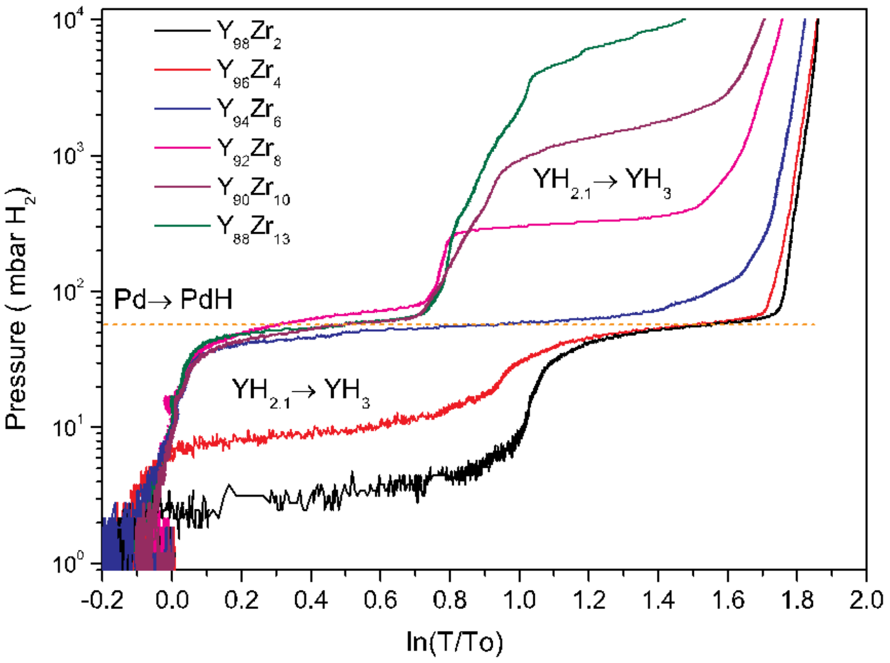

Supplementary Figure 4: Room temperature PTIs of 1 x 1 cm Y-Zr thin films with uniform thickness (60 nm Y_xZr_{1-x} - 5 nm Ti - 30 nm Pd) but different Zr concentration as indicated. It is interestingly to note that the plateau hydrogen pressure for Pd → PdH_x is same (around 40 mbar) for all the samples while that for $YH_2 \rightarrow YH_3$ is clearly dependent on the Zr concentration. Note that unlike the gradient samples, the magnitude of $\ln(T/T_o)$ (change in transmittance upon hydrogenation) does not vary much for the uniformly thick samples. The minor decrease in $\ln T/T_o$ with increasing Zr concentration is due to the fact that the ZrH_x phase is metallic and does not contribute to the change in transmittance.

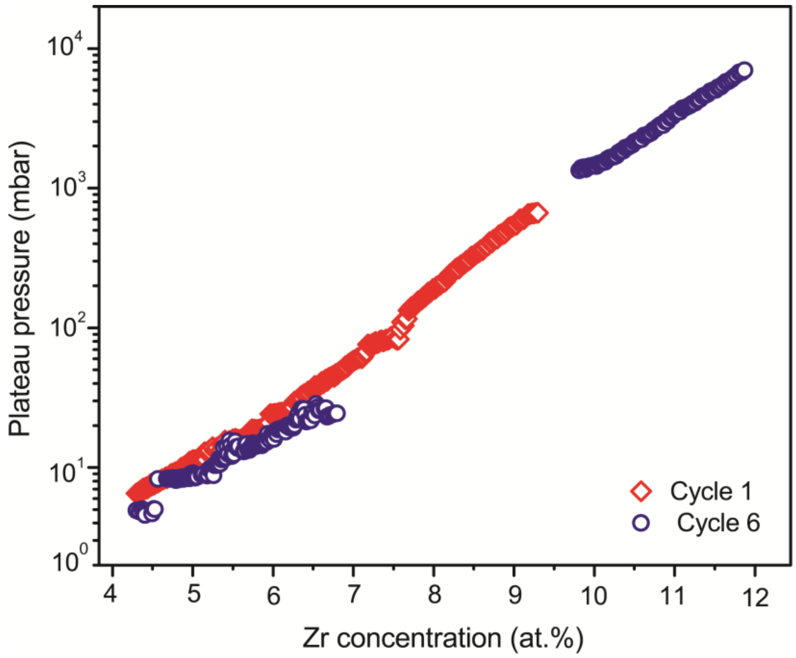

Supplementary Figure 5: Relationship between the equilibrium hydrogen pressure for the $YH_2 \rightarrow YH_3$ plateau and the Zr concentration as measured at room temperature. The figure shows a logarithmic relationship between the Z concentration and the plateau pressure. To minimize error due to the logarithmic increase in pressure during hydrogenation, different pressure range were used to obtain a more accurate plateau pressure for different Zr concentration range along the gradient sample. It can be seen that the relationship between Zr concentration and plateau pressure is largely the same in the 1st and 6th hydrogenation cycles, irrespective of the concentration range measured along the sample.

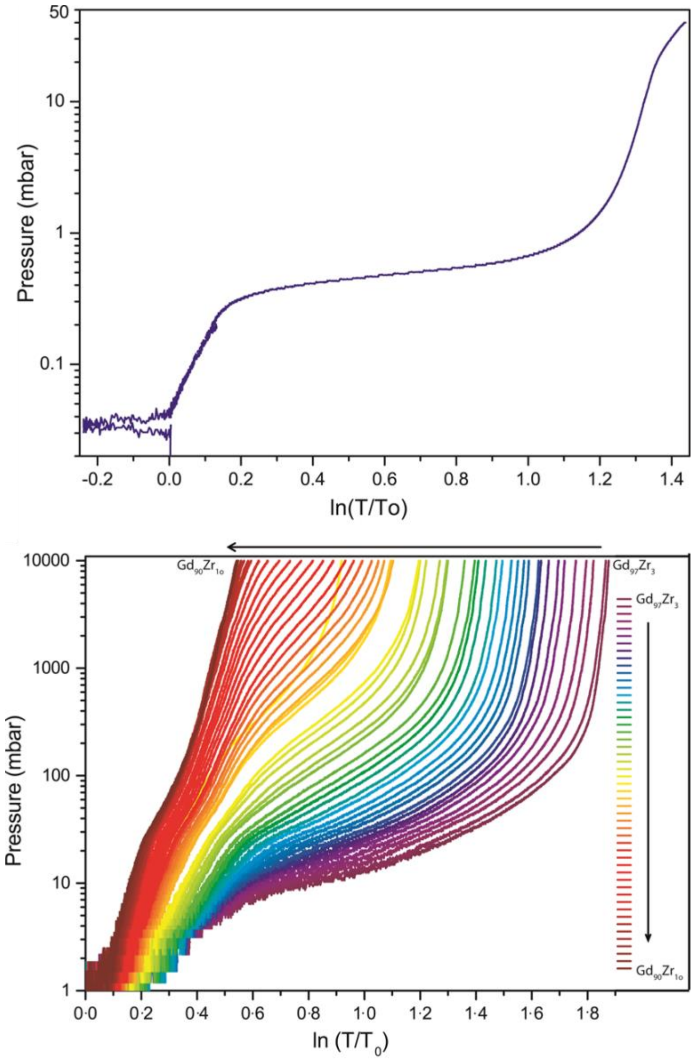

Supplementary Figure 6: Room temperature pressure transmission isotherms for gadolinium-hydrogen system during the third hydrogen absorption cycle. The top figure shows the PTI for a pure Gd thin film (i.e. 60 nm Gd - 5nm Ti – 10 nm Pd) while the bottom shows the PTIs for Zr doped Gd i.e. a gradient sample with composition $Gd_{97}Zr_3$ – $Gd_{10}Zr_{90}$. It can be seen that similar to the $YH_2 \rightarrow YH_3$, the plateau hydrogen pressure for the $GdH_2 \rightarrow GdH_3$ phase transition is clearly dependent on the Zr concentration. The figure displays the PTI's of 53 position along the 70 mm sample, hence each line or step corresponds to 0.15 atom% increase in Zr concentration. Compared to Y, the Gd-H system exhibits a more tilted isotherm when measured as same the conditions. The darkening effect due to $GdH_{1.9} \rightarrow GdH_{2.1}$ can be observed in pure Gd around 0.03 mbar H_2. Note also that it is the 3rd hydrogen absorption cycle that is displayed in the figure.

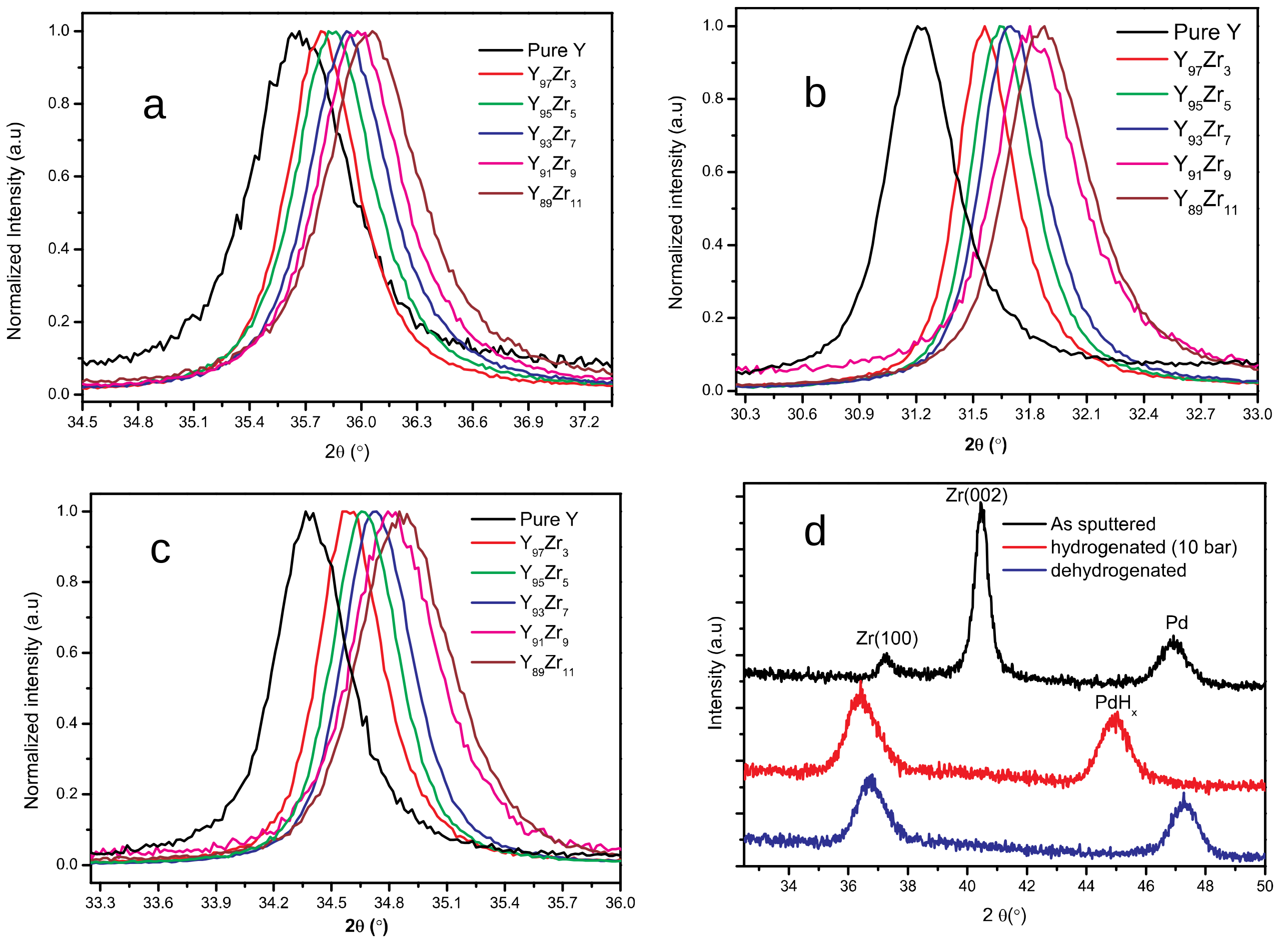

Supplementary Figure 7: XRD pattern for Y_xZr_{1-x}-Ti-Pd multilayer thin film with varying Zr concentrations. Figures a-c shows the Y (002) diffraction peak in the as prepared state or metallic Y (a), fully hydrogenated state or YH_3 phase (b) dehydrogenation or YH_2 phase (c). The figures shows that that the 2θ value of the Y (002) diffraction peak increases with increasing Zr concentration due to the compression of the YH_x lattice (decreasing in d-spacing). Interesting this lattice compression is observed in all the YH_x phases, showing that the (de)hydrogenation process does not destroy the compression/clamping effect. Figure d shows the Zr-Ti-Pd (60-5-15 nm respectively) thin film in the as prepared, hydrogenated and dehydrogenated states. The figure shows that pure hcp Zr thin film is converted to tetragonal (ε-$ZrH_{1.7\text{-}2}$) phase after hydrogenation (10 bar), while cubic zirconium hydride is observed after dehydrogenation.

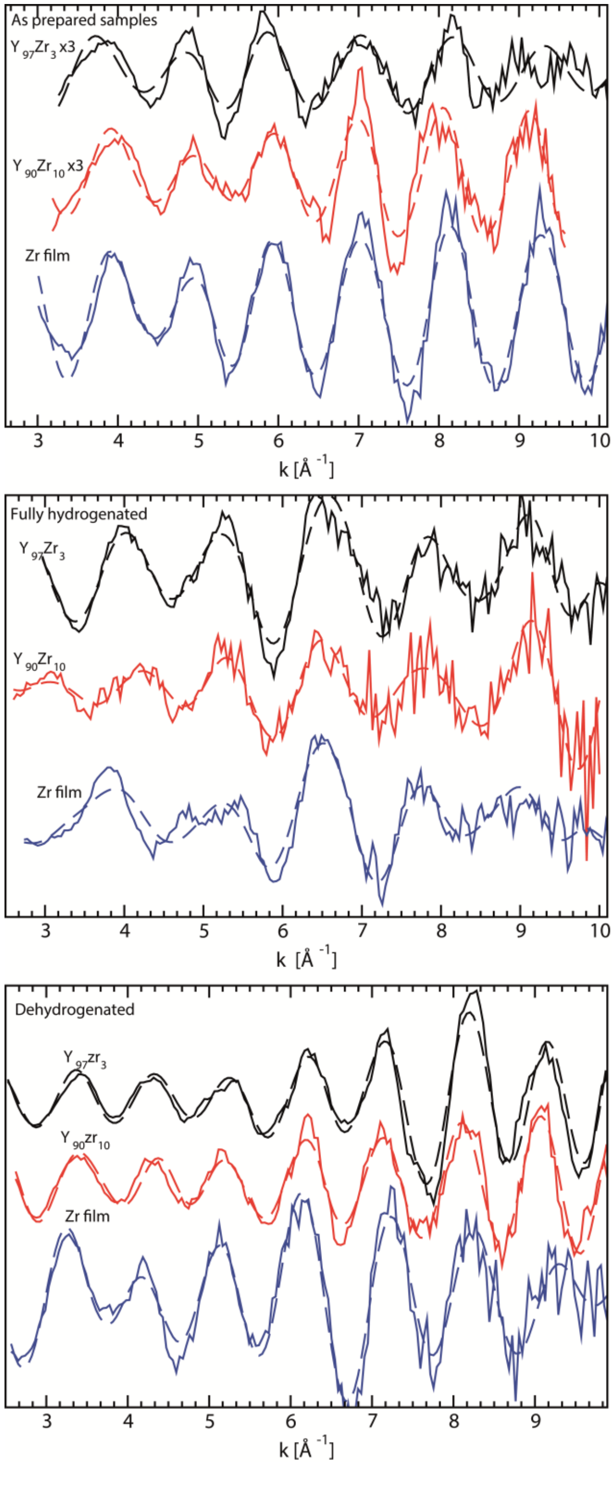

Supplementary Figure 8. Experimental and simulated Zr-K- edge EXAFS data for the samples in the as-prepared, fully hydrogenated and dehydrogenated states. The fits were based on the parameters in Supplementary Table 2.

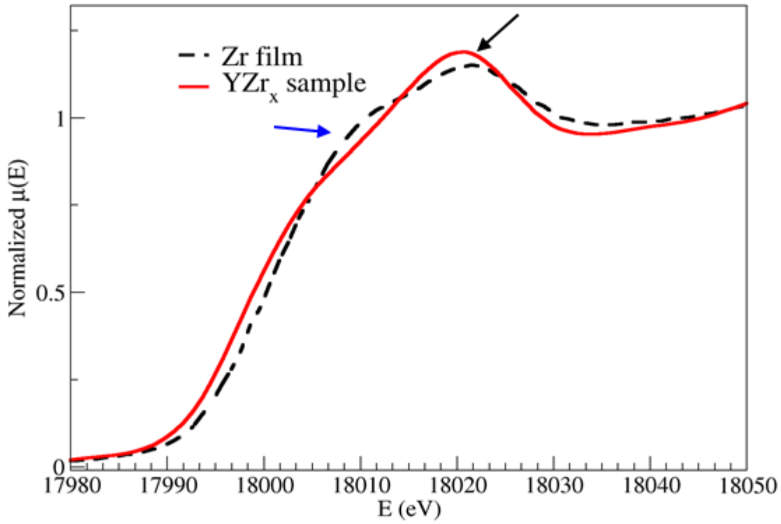

Supplementary Figure 9. XANES spectra of ZrHx film (dashed black line) and $YZrH_x$ sample (red line). The arrows highlight the differences between the two hydrogenated samples.

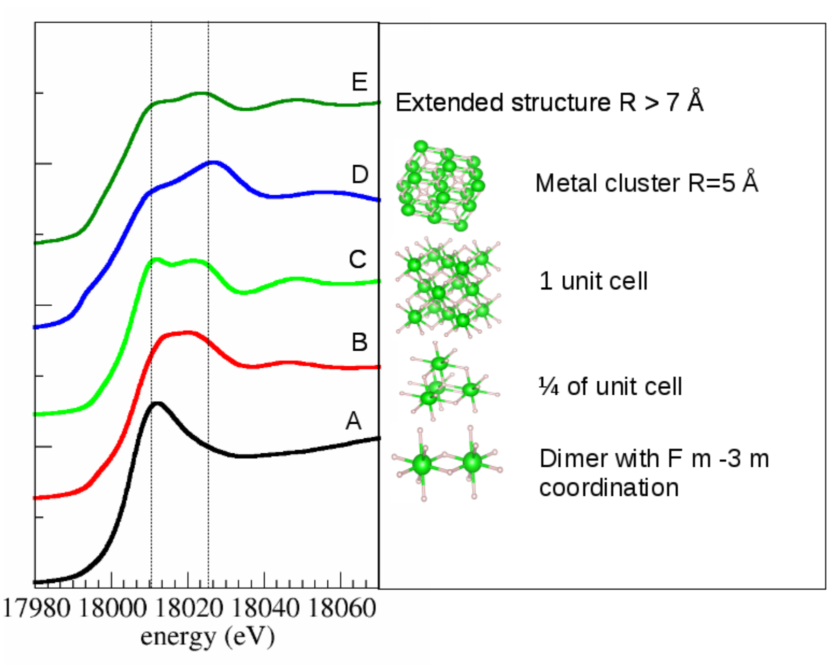

Supplementary Figure 10. Simulated XANES signals of the cubic structural arrangement (Fm -3 m) of the different clusters shown aside.

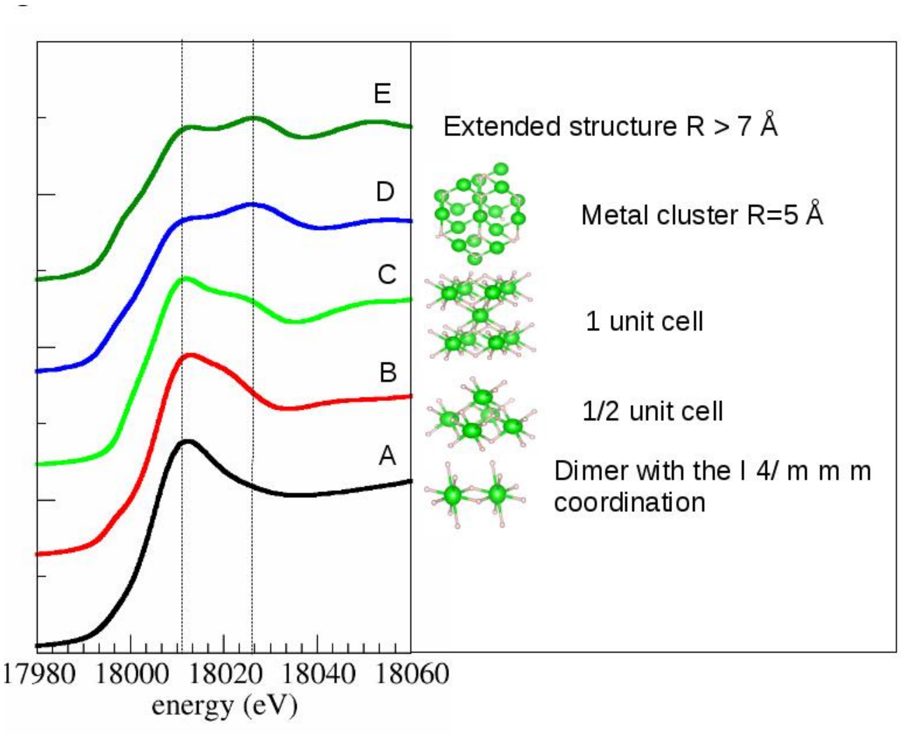

Supplementary Figure 11. Simulated XANES signals of the tetragonal structural arrangement (I 4/m m m) of the different clusters shown aside.

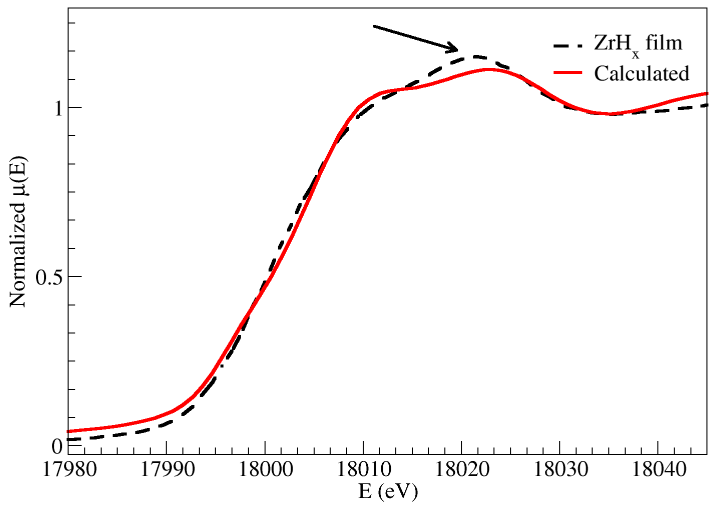

Supplementary Figure 12. A combination of cubic and tetragonal phases gives the best fit to the measured spectra of ZrH_x films. The arrow points the main misfit between the calculated and observed data.

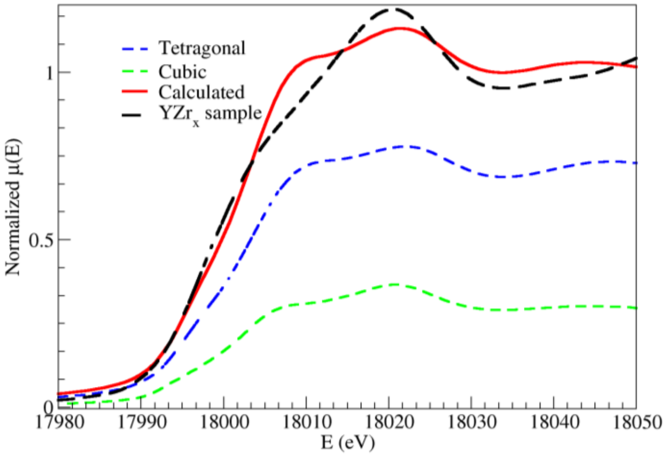

Supplementary Figure 13. Measured XANES (dashed black line) of $YZrH_x$ samples versus best simulation (continuous red line) of the YH_x film. For a comparison, the cubic (dashed green line) and tetragonal (dashed blue line) components are reported.

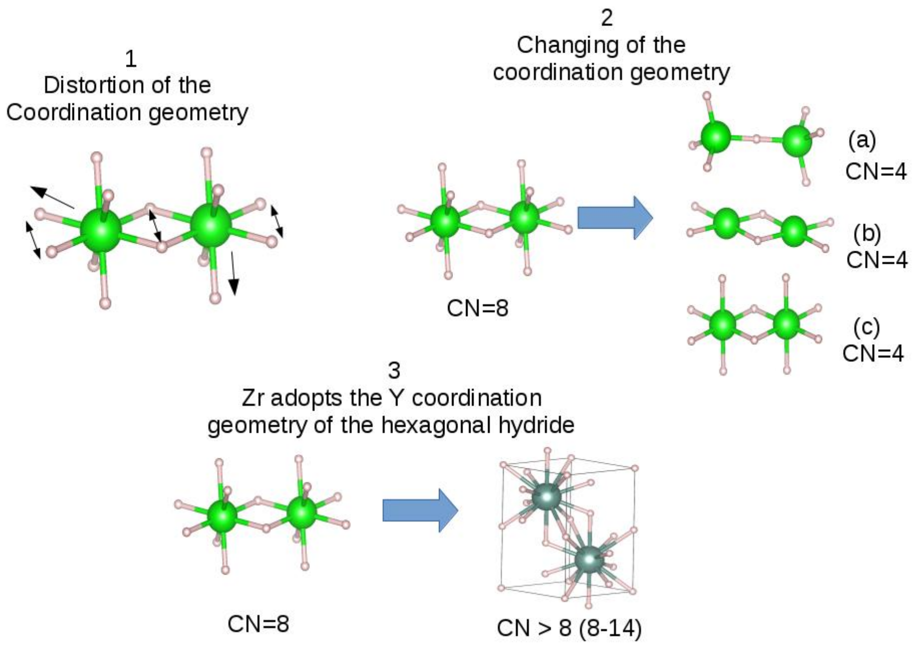

Supplementary Figure 14. Different strategies used to simulate XANES spectra of $YZrH_x$ sample.

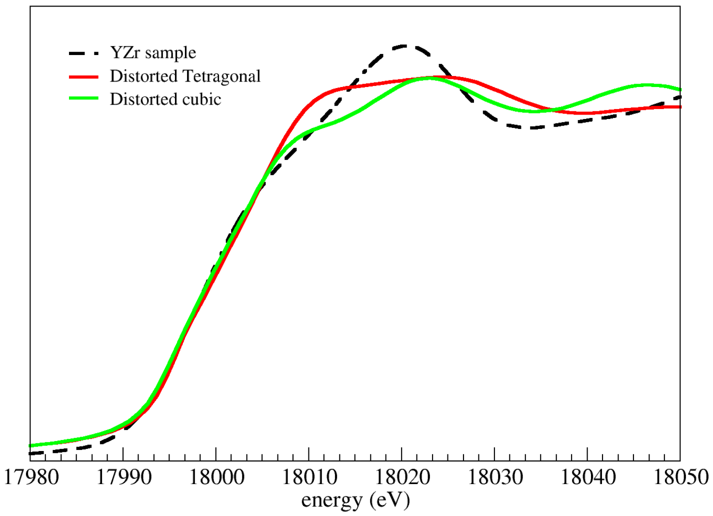

Supplementary Figure 15. Effect of the distortion in the cubic and tetragonal geometry on the calculated XANES.

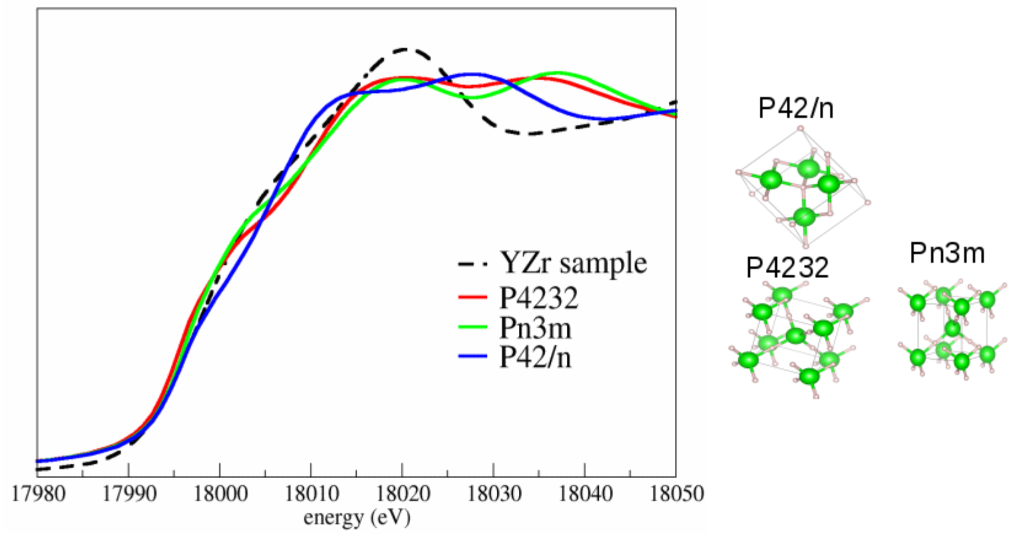

Supplementary Figure 16. Different crystallographic structures which adopts the coordination geometry discussed in Supplementary Figure 14.

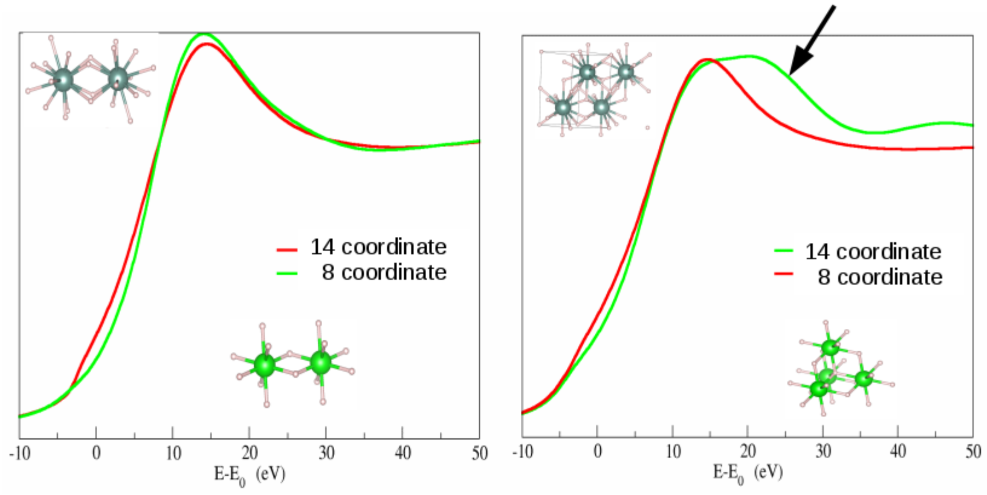

Supplementary Figure 17. XANES simulations results for ZrH_2 clusters with 12 coordinate Zr vs 8 coordinate Zr

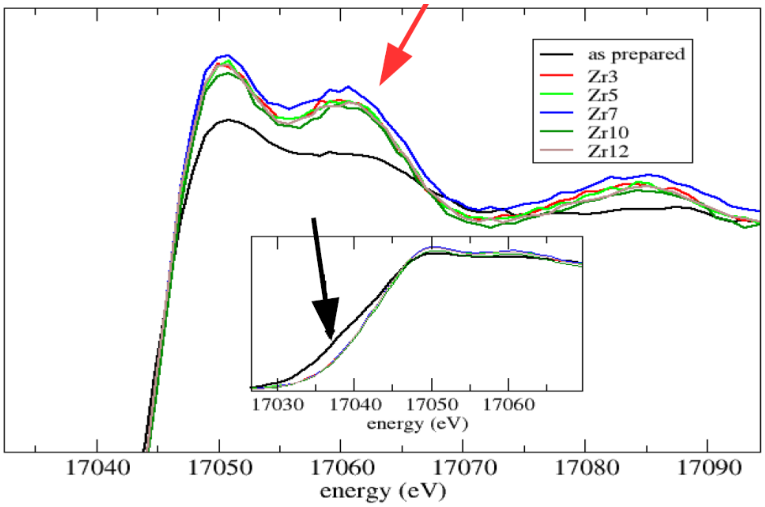

Supplementary Figure 18: The XANES spectra of the Y-K edge of Y-Zr films with different Zr concentration (atomic % as indicated on the figure) in the as-prepared and dehydrogenated states. The black arrow in the inset indicates the variation of the edge position (2 eV) between the as-prepared (Y) and the dehydrogenated (YH_2) states. The red arrow marks the main modification in the spectra of the dehydrogenated samples as a function of the Zr concentration

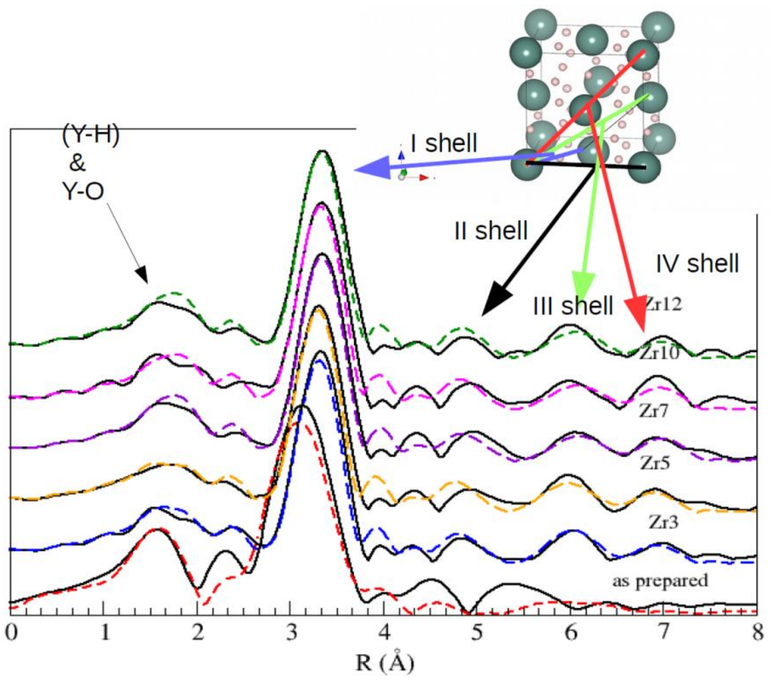

Supplementary Figure 19. Fourier transform (FT) of the EXAFS data for samples in the supplementary Figure 17. The detailed fitting shows that the as-prepared and dehydrogenated samples have a hexagonal and cubic packing arrangement respectively as expected based on the XRD results. Interesting the lattice parameters were found to decrease slightly with increasing Zr contents as shown below, and in line with the XRD results.

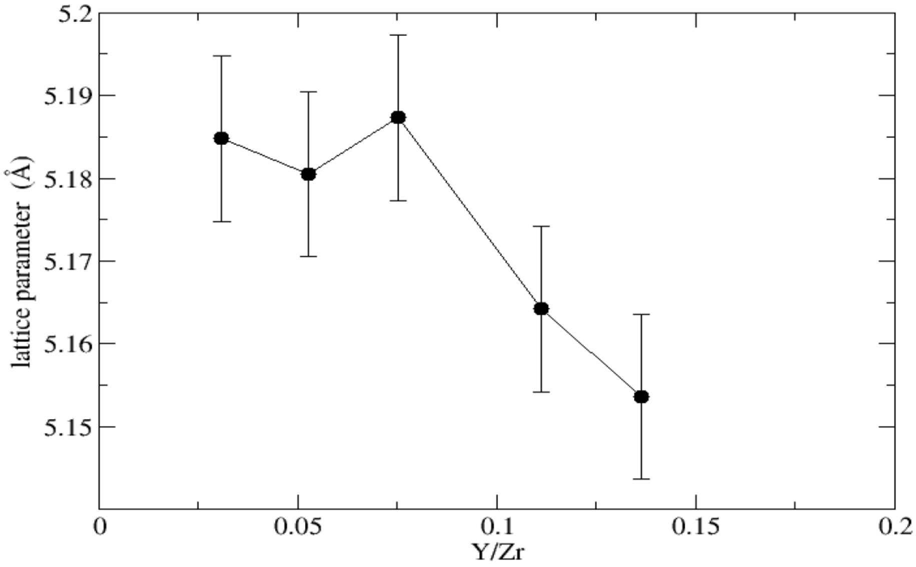

Supplementary Figure 20. Lattice parameter of the different YZr samples as obtained from the detailed EXAFS data analysis (with the error bar= 0.01) versus the Zr/Y ratio

Supplementary Tables

Supplementary Table 1: d-spacing (in Å) as a function of Zr concentration (atomic %) as derived from XRD measurements

Sample	As prepared (Y)	Hydrogenated (YH_3)	Dehydrogenated (YH_2)
Y	2.921	3.312	3.038
$Y_{97}Zr_3$	2.904	3.284	3.015
$Y_{95}Zr_3$	2.900	3.270	3.012
$Y_{93}Zr_7$	2.895	3.256	3.009
$Y_{91}Zr_9$	2.890	3.248	3.004
$Y_{89}Zr_{11}$	2.882	3.237	2.999

Supplementary Table 2: Results from Zr K-edge EXAFS Analysis.
Superscript *c* indicates hydride cubic phase SG 225; Superscript *t* indicates hydride tetragonal phase SG 139 *a*. N_i are the coordination numbers, R_i are the Zr-M (Zr or Y) distance of the different crystallographic phases needed to fitting the data. σ_i are the Debye Waller factors. The parameters errors are in the last digit.

	**Zr**	**$Y_{85}Zr_{15}$**	**$Y_{90}Zr_{10}$**	**$Y_{93}Zr_7$**	**$Y_{97}Zr_3$**
			**As prepared**		
**N**1^{hcp}	8.8	3.0	3.0	3.2	3.5
**R**1^{hcp} **(Å)**	3.21	3.14	3.02	3.20	3.16
**σ**1^{hcp}**(Å^2)**	0.008	0.008	0.008	0.007	0.008
			**Hydrogenated**		
**N**1 (Zr-H)	7.5	7.0	6.9	8.0	7.00
**R**1 (Zr-H) **(Å)**	1.85	1.98	1.98	1.87	1.88
**σ**1 (Zr-H) **(Å^2)**	0.0	0.	0.	0.	0.
**N**2 (Zr-Zr)^c	5.	-	-	-	-
**R**2 (Zr-Zr)^c **(Å)**	3.35	-	-	-	-
**σ**2 (Zr-Zr)^c **(Å^2)**	0.008	-	-	-	-
**N**2 (Zr-Zr)^t	8.0	5.0	5.5	7.	7.
**R**2 (Zr-Zr)^t **(Å)**	2.95	2.86	2.90	2.88	2.86
**σ**2 (Zr-Zr)^t**(Å^2)**	0.0094	0.007	0.007	0.008	0.008
**% Cubic**	31%	0%	0%	0%	0%
			**Dehydrogenated**		
**N**1 (Zr-H)	4.1	4.0	3.0	4.0	4.0
**R**1 (Zr-H) **(Å)**	2.03	1.98	1.98	2.02	2.03
**σ**1 (Zr-H) **(Å^2)**	0.	0	0	0	0
**N**2 (Zr-Zr)^c	4.	6.	5.	6.	6.
**R**2 (Zr-Zr)^c **(Å)**	3.34	3.59	3.41	3.49	3.39
**σ**2 (Zr-Zr)^c **(Å^2)**	0.008	0.008	0.007	0.009	0.009

Supplementary Table 3*:* Results from Y K-edge EXAFS Analysis comparing Y and $Y_{90}Zr_{10}$ in the as-prepared, hydrogenated and dehydrogenated states.

N_i are the coordination numbers. Ri are the distances of the different shells and are reported in Å; σ_i are the Debye Waller factors (the disorder terms) reported in Å^2. In the cubic structure N_1 is the Y-H and N_2 is Y-Y distance respectively. In the Hexagonal, N1 is the Y-H, N2 is the first Y-Y whereas N3 is the Y-Y distances of the first shell respectively. The parameters errors are in the last digit. W^{hcp} is the amount of hexagonal phase (in %) present in the sample. Note that the presence of minor fraction of cubic phase in the as-prepared state is in line with the fact that metallic Y target often contain small amounts of hydrogen (YH_2) impurities.

	**Y As-prepaered**	**Y Hydrogenated**	**Y Dehydrogenated**	**$Y_{90}Zr_{10}$ As-prepaered**	**$Y_{90}Zr_{10}$ Hydrogenated**	**$Y_{90}Zr_{10}$ Dehydrogenated**
$N1^{cub}$	3.0	-	4.	3.0	-	5
$R1^{cub}$	2.274	-	2.24	2.25	-	2.21
$\sigma1^{cub}$	0.009	-	0.004	0.009	-	0.012
$\theta1^{cub}$	109	-	109	109	-	109
$N2^{cub}$	5.	-	9.	4.5	-	6.
$R2^{cub}$	3.67	-	3.37	3.69	-	3.65
$\sigma2^{cub}$	0.017	-	0.007	0.015	-	0.017
$N1^{hcp}$	9.	9.	-	9.	7.	6.
$R1^{hcp}$	2.49	2.531	-	2.30	2.401	2.437
$\sigma1^{hcp}$	0.015	0.014	-	0.015	0.009	0.010
$N2^{hcp}$	4.	4.	-	4.0	5.	4.
$R2^{hcp}$	3.69	3.72	-	3.70	3.65	3.69
$\sigma2^{hcp}$	0.016	0.025	-	0.016	0.010	0.009
$N3^{hcp}$	4.	4.	-	4.0	5.	4.
$R3^{hcp}$	3.98	3.99	-	3.99	3.99	3.85
$\sigma3^{hcp}$	0.022	0.025	-	0.022	0.012	0.028
W^{hcp}	70%	100%	0%	70%	100%	20%

Supplementary Table 4*:* Results from Y K-edge EXAFS Analysis of dehydrogenated $YZrH_x$ films with varying Zr concentrations. The M-CN is a factor representing the multiplicity of the coordination numbers . Note that for this fully dehydrogenated samples, only the cubic (YH_2) phase is observed.

Parameter	**Y**	**$Y_{97}Zr_3$**	**$Y_{95}Zr_5$**	**$Y_{93}Zr_7$**	**$Y_{90}Zr_{10}$**	**$Y_{88}Zr_{12}$**
**N**$1_{Y\text{-}H}$	4.0	4.4	4.7	4	4.2	4.2
**R**$1_{Y\text{-}H}$	2.239	2.24	2.23	2.24	2.25	2.25
$\sigma 1_{Y\text{-}H}$	0.004	0.004	0.003	0.004	0.005	0.005
**M-CN**	0.7	0.68	0.72	0.71	0.71	0.71
**a** $_{Y\text{-}Y}$	5.154	5.185	5.181	5.188	5.164	5.154
$\sigma 1_{Y\text{-}Y}$	0.007	0.006	0.005	0.005	0.007	0.006
$\sigma 2_{Y\text{-}Y}$	0.006	0.009	0.007	0.008	0.006	0.008
$\sigma 3_{Y\text{-}Y}$	0.010	0.12	0.010	0.011	0.10	0.11
$\sigma 4_{Y\text{-}Y}$	0.015	0.014	0.017	0.015	0.015	0.015

Supplementary Methods

Zr-XANES simulations

The measured XANES spectra of the hydrogenated ZrH_x and $YZrH_x$ samples are shown in **Supplementary Figure 9**. Important differences can be observed: i) the shoulder at 18010 eV present in the ZrH_x film (blue arrow) is shifted towards lower energy in the $YZrH_x$ sample, and is less pronounced than for the ZrH_x film; ii) The intensity of the main absorption peak (indicated by the black arrow) is higher for the $YZrH_x$ film than the ZrH_x film. These differences suggest a strong modification of the coordination geometry of the Zr in the $YZrH_x$ films with respect to the ZrH_x film.[2,3]

In order to asses these changes, XANES simulations have been performed using FDMNES package program. According to the code, the theoretical signals are simulated using the multiple scattering theory in Green formalism and based on the muffin-tin approximation on the potential shape. In the simulated signals, the muffin-tin radii have been tuned to have a 10% overlap between the different spherical potentials. To correct for inelastic losses, the Hedin–Lundqvist exchange potential has also been used. The approximation of non-excited absorbing atoms, which better reproduces the experimental data, has been adopted. The theoretical XANES spectra were calculated considering that all the atoms are surrounding the absorber Zr within a 7 Å radius sphere. In addition, in the simulation of the XANES spectra the tabulated core-hole broadening together with an energy resolution of 1.9 eV have been set. The effect of the structural disorder has not been considered in the calculation of the theoretical signal. The XANES spectra, three scans per sample, have been energy-calibrated, averaged and then compared with the calculated spectra. Since the Y and Zr atoms differ from one electron, the fluorescence Kα lines are close each other. Due to the high amount of the Y atoms with respect to the Zr in the samples, self-absorption correction of the XANES data recorded at Zr K edge is necessary.

Results for the ZrHx films

In **Supplementary Figure 10 and 11**, we show the results from simulations for cubic and tetragonal structures of different ZrH_x cluster sizes. It is not surprising that both structures have a

similar XANES signal because Zr has the same eight fold coordination geometry in both of these atomic arrangements. Notably, both cubic and tetragonal structures have a shoulder at 18010 eV, whose intensity is a function of the cluster size. On the other hand, the ratio of the two main peaks, indicated by the dashed lines in the figures, is correlated with the different atomic arrangements.

As shown in **Supplementary Figure 12**, using only the tetragonal structure is not sufficient to reproduce the intensity of the second peak at 18030 eV of the Zr film spectrum. Therefore based on the EXAFS results discussed in the main paper, to get a better agreement with the measured XANES spectrum, the simulation needed both a tetragonal and a cubic phase.

$YZrH_x$ films

The conclusion drawn for the ZrH_x film is not valid for the $YZrH_x$ samples. **Supplementary Figure 13** shows that neither cubic, tetragonal, nor a mixture of these two phases, is able to reproduce the XANES spectrum of the fully hydrogenated YZr ($YZrH_x$) samples. In order to model the data three possible scenarios, summarized in **Supplementary Figure 14**, are possible: 1) the cubic or the tetragonal Zr coordination geometry is highly distorted; 2) the coordination geometry of the Zr is different from the two mentioned main zirconium hydride structures, because other hydride structures are possible;[4-6] 3) Strong interaction with Y induces a modification of the coordination geometry. Interestingly, the distortion introduced by energy relaxing the bonded atoms,[6] in the eight fold Zr is not sufficient to reproduce the features observed in the $YZrH_x$ sample. The result is displayed in **Supplementary Figure 15**. This conclusion is also drawn for the possible (a) and (b) Zr coordination geometries shown in **Supplementary Figure 14** as proposed in previous studies.[4,5] These results are reported in **Supplementary Figure 16**. It is worth noticing that the octahedral coordination geometry (c) was not considered because it has never been reported in literature.

The possibility that the Zr adopts the same coordination of the Y in the hydride is now examined. Based on the Y coordination geometry shown in **Supplementary Figure 14**, three main Y-H distances can be noticed: 6 Y-H at 2.27 Å, 2 Y-H at 2.49 Å and 6 Y-H at 2.69 Å respectively. It is worth noticing that the possibility to have this different coordination geometry suggests that Zr

can have also a bigger coordination number.[4] **Supplementary Figure 17** shows a comparison of the calculated XANES for the 8-fold and 14-fold coordination zirconium hydride. As shown in the figure, different coordination geometry can cause strong difference in the growth of the clusters. So, four Zr atoms in a cubic packing produce already the feature which characterizes the corresponding XANES spectrum. Therefore, as a first attempt, the possibility to have the ZrH_x only in hexagonal packing was considered. To this aim in order to generate the ZrH_x cluster, the hexagonal lattice parameters have been derived according to the distances obtained by the EXAFS analysis. As expected the shoulder at 18009 eV, characterizing the cubic and tetragonal atomic arrangement is absent. However, other differences are present in the simulated signal, which allow to exclude having the Zr in the pure hexagonal structure of the yttrium hydride.

The other possibility was to consider the Zr with the same coordination geometry used so far for the hexagonal packing, but within a distorted structure. To obtain such a cluster, the hexagonal atomic arrangement has been relaxed with a Monte Carlo algorithm which progressively relaxes the bonded structure.[6] This relaxed structure, after a standard minimization of the energy according to SCF procedure available in FDMNES package[7], has been used as input for the XANES calculations. These produced a very good fit to the data as shown in **Figure 4** in the main paper. In conclusion, the XANES simulation shows that the Zr in the ZrH_x thin films exists in tetragonal and cubic phases. The weight percentage of the two phases agrees with the EXAFS results. However for the $YZrH_x$ films, the Zr coordination geometry is neither cubic nor tetragonal but it seems to adopt a configuration which fits the hexagonal YH_3 environment. Hence, the Zr changes its coordination geometry from eight fold to higher value up to 14. However, due to the strong poly-dispersion of the system, it is not trivial to establish the exact coordination number of the Zr.

Y K- edge EXAFS and XANES Analysis

For the Y K-edge we assumed that the YH_2 clusters have a cubic (Fm-3m) structure, while the Y and YH_3 are hexagonal as reported in literature. For the cubic, γ^2 terms take into account the first and second coordination shell, respectively. The first-shell Y−Y distance is linked to the fcc a lattice parameter by R1 = a /(2)1/2 , while the second neighbors are placed at R2 = **a**. The

higher shells are calculated according to three-body contributions: the third-shell term, at R3 = **a** (3)1/2 /2, is relative to an isosceles triangle with two first neighbor R1 sides and vertex angle θ = 120°, whereas the fourth shell contribution, involving particularly strong multiple scattering contributions, is obtained from the degenerate (θ =180°) triangle formed by three aligned first neighbors. Then, only the lattice constant **a** , the Debye Waller factors σ_i (i=1-4) and the one factor which takes into account the cluster size, are the parameters needed for the structural analysis for the YH_2 clusters. This allows a limited number of fitting parameters. For the hexagonal structures(Y and $YH_{3)}$, the second shell Y-Y is correlated to the first by considering the three body configurations involving the angle at 60°. The main results are summarized in Supplementary figures 18-20 and supplementary Table 3-4

Supplementary figure 18 shows the XANES spectra of the Y k-edge of the as-prepared and dehydrogenated samples with various Zr concentration. The as-prepared samples show very similar spectra irrespective of the Zr concentration, therefore for clarity, we included just one of the spectra. The similarities of the XANES spectra of the as-prepared samples points once gain to the fact that Y did not alloy with Zr, since the electronic structure is unchanged. However after hydrogenation and dehydrogenation, it is clear that the edge position of the XANES spectra differs by about 2 eV, and the main features are different as shown by the arrows in the figure. The change in the spectra is due to change in the electronic properties and structure of Y upon hydrogen absorption (Y versus YH_2).

Supplementary References

1 Mooij, L., Baldi, A., Boelsma, C., Shen, K., Wagemaker, M., Pivak, Y., Schreuders, H., Griessen, R. & Dam, B. Interface energy controlled thermodynamics of nanoscale metal hydrides. *Advanced Energy Materials* **1**, 754-758 (2011).

2 Bugaev, A., Guda, A., Lomachenko, K., Lazzarini, A., Srabionyan, V., Vitillo, J., Piovano, A., Groppo, E., Bugaev, L. & Soldatov, A. in *Journal of Physics: Conference Series.* 012032 (IOP Publishing).

3 Bugaev, A. L., Guda, A. A., Lazzarini, A., Lomachenko, K. A., Groppo, E., Pellegrini, R., Piovano, A., Emerich, H., Soldatov, A. V. & Bugaev, L. A. In situ formation of hydrides and carbides in palladium catalyst: When XANES is better than EXAFS and XRD. *Catal. Today* **283**, 119-126 (2017).

4 Christensen, M., Wolf, W., Freeman, C., Wimmer, E., Adamson, R. B., Hallstadius, L., Cantonwine, P. E. & Mader, E. V. H in α -Zr and in zirconium hydrides: solubility, effect on dimensional changes, and the role of defects. *J. Phys.: Condens. Matter* **27**, 025402 (2015).
5 Zhu, W., Wang, R., Shu, G., Wu, P. & Xiao, H. First-principles study of different polymorphs of crystalline zirconium hydride. *The Journal of Physical Chemistry C* **114**, 22361-22368 (2010).
6 CrystalMaker(R).
7 Joly, Y. X-ray absorption near-edge structure calculations beyond the muffin-tin approximation. *Phys. Rev. B.* **63**, 125120 (2001).
