# Supplementary material for: Metal-hydrogen systems with an exceptionally large and tunable thermodynamic destabilization
Source: Nat Commun. 2017 Nov 29;8:1846. doi: 10.1038/s41467-017-02043-9 (PMC5705672; doi:10.1038/s41467-017-02043-9)
Supplement: Supplementary file 3 — Description of Additional Supplementary Files [file 41467_2017_2043_MOESM3_ESM.docx]

**Description of Additional Supplementary Files**

File Name: Supplementary Movie 1

Description: Hydrogenation of an YZr “hydrogen thermometer”: The movie shows the color change associated with the first cycle hydrogenation of YZr gradient thin films at room temperature (~25 °C). The topmost sample contains 0-2 atomic % Zr (Y-Y_98_Zr_2_), the sample in the middle contains 1.5 - 9% Zr (Y_98_Zr_1.5_-Y_91_Zr_9_) while the bottom sample contains 3.4-14.5% Zr (Y_96.6_Zr3._4_- Y_85.5_Zr_14.5_). The hydrogen pressure was increased logarithmically from 10^-1^ -10^3^ mbar in 10 hours and the image of the samples acquired every 10 seconds.

File Name: Supplementary Movie 2

Description: Dehydrogenation of an YZr “hydrogen thermometer”: Color change associated with the dehydrogenation of YZrH_3_ gradient thin films in the presence of oxygen at room temperature (~25 °C). The topmost sample contains 0-2 atomic % Zr (Y-Y_98_Zr_2_), the sample in the middle contains 1.5-9% Zr (Y_98_Zr_1.5_-Y_91_Zr_9_ while the bottom sample contains 3.4-14.5% Zr (Y_96.6_Zr3._4_ - Y_85.5_Zr_14.5_).

File Name: Supplementary Movie 3

Description: Second cycle hydrogenation of an YZr “hydrogen thermometer”: Color change associated with the second cycle hydrogenation of YZr gradient thin film at room temperature (~25 °C). The topmost sample contains 0-2 atomic % Zr (Y-Y_98_Zr_2_), the sample in the middle contains 1.5-9 % Zr (Y_98_Zr_1.5_-Y_91_Zr_9_ while the bottom sample contains 3.4-14.5% Zr (Y_96.6_Zr_3.4_ - Y_85.5_Zr_14.5_). The pressure was increased logarithmically from 10^-1^ -10^4^ mbar in 15 hours and the image of the samples acquired every 10 seconds
